# Long-Term Survival of Biliary Atresia without any Surgery: Lessons Learnt from Lamprey

**Published:** 2014-04-01

**Authors:** V Raveenthiran

**Affiliations:** Department of Pediatric Surgery, SRM Medical College and Hospital SRM University, Chennai, India.

The greatest leap in human evolution is the ability of vertebrate cells to handle cholesterol metabolism more effectively than invertebrates. This resulted in the development of features - such as greater regulation of trans-membranous cell transport, synthesis of steroid hormones and myelinic insulation of nerves - which are critical for higher evolution. The increased need of cholesterol synthesis also necessitated the evolution of hepatobiliary system for better elimination of unwanted cholesterol and its metabolites. Stray misfiring of this complex development leads to problems such as biliary atresia (BA).

Athena is frequently awed by the clinical care of BA. Although Morio Kasai’s ingenious operation was a breakthrough, long-term cure is still elusive. [1, 2] Eventually more than 60% of the patients require a liver transplantation, which is frequently unaffordable, unavailable and impractical. [3] Further, the long-term outcome of liver transplants in BA is currently uncertain. [4, 5, 6] Athena is upset that science is really stuck at this impasse. She used to wonder as to why we do not think beyond surgery for the cure of this defiant problem. Her concern is addressed by a recent series of studies on the biliary system of sea lampreys. Lampreys develop biliary atresia during the normal process of their metamorphosis, yet they do not suffer liver injury like human infants. [7] They maintain normal serum and tissue levels of bile salts and bile pigments despite BA [8, 9] and they continue to live adult life. They not only survive adulthood but also exponentially grow, that their body mass increases 500 fold in 2 years. [10] Discovering the adaptive mechanism of lampreys may hold the key for successful management of BA in human newborns. 

**Life-cycle of lampreys**

Lampreys are eel-like primitive vertebrates. (Figure 1) They belong to the superclass agnatha (jawless fishes). Among the 38 different species of lampreys, the biliary system of sea lamprey (Petromyzon marinus) has been extensively studied. The life-cycle of lampreys consists of an immature larval phase (ammocoetes) and a sexually matured adult phase. [11] Larvae hatch from eggs laid in fresh water streams. The saprophytic larvae dwell in mud-burrows and feed on decaying organic debris. After a variable period of 4 to 21 years, the larvae metamorphose into sexually matured adult male and female. [10, 11] During metamorphosis, if maturation is protracted, the resultant adults become non-parasitic (fluvial type); they remain in fresh water streams, spawn and quickly die. [11] The rest of the adults develop into parasitic lampreys (anadromous type). These parasitic lampreys migrate to sea or lakes and derive their nutrition by parasitic attachment to fishes. After 2 to 3 years, the parasitic lampreys return to fresh water for spawning and they subsequently die. 

**Figure:**
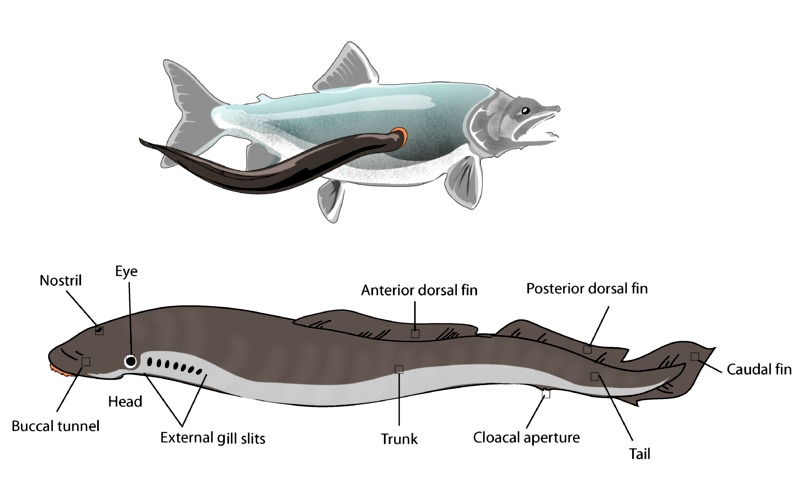
Figure 1: Diagram showing the external morphology of sea lamprey (lower panel) and its parasitic attachment to sea fish (upper panel). (Source of diagram Wikipedia - available under creative commons).

**Biliary Atresia Model**

Larvae of lamprey have well developed hepatobiliary system. [12] The liver cells are arranged in sinusoidal pattern similar to human liver. Intrahepatic bile ducts (IHBD) drain into common bile duct (CBD), which in turn drains into the intestine. These ducts are lined by columnar or cuboidal epithelium similar to that of human beings. The biliary system also consists of an intrahepatic gall bladder and cystic duct, which joins the main CBD. Portal triad, central vein, hepatocyte architecture and enterohepatic circulation of larval lamprey are remarkably similar to that of human beings. [13, 14] However, during metamorphosis, the biliary system completely disappears mimicking the congenital variety of human BA. Probably due to this major structural alternation, non-parasitic adult lampreys stop feeding at the start of metamorphosis. They spawn and die without resuming feeding. These non-parasitic lampreys are of no great significance to pediatric surgeons. Conversely, the parasitic type of lampreys interest Athena as they could live their full adult life despite BA. 

Youson identified 7 stages of larval metamorphosis. [7] Atretic changes of the biliary system were previously thought to start from stage-2 and complete by stage-6. However, Boomer et al [15] have recently shown that even in stage-1 sub-cellular DNA fragmentation heralds the onset of degeneration. (Table 1) DNA fragmentation eventually leads to cell apoptosis and macroscopic degeneration of bile ducts. [16] Gross and microscopic morphological changes of the bile ducts of metamorphosing lamprey astonishingly mimic that of BA in human beings. [7] The entire metamorphic disappearance of biliary apparatus takes 1 to 3 months. [10] Athena is amused by the coincidence of this 3-months’ duration because in human BA the prognosis is better when porto-enterostomy is done within 3 months of birth. These similarities embolden us to safely draw parallels between the BA of the two species and extrapolate the conclusions and principles. 

**Figure:**
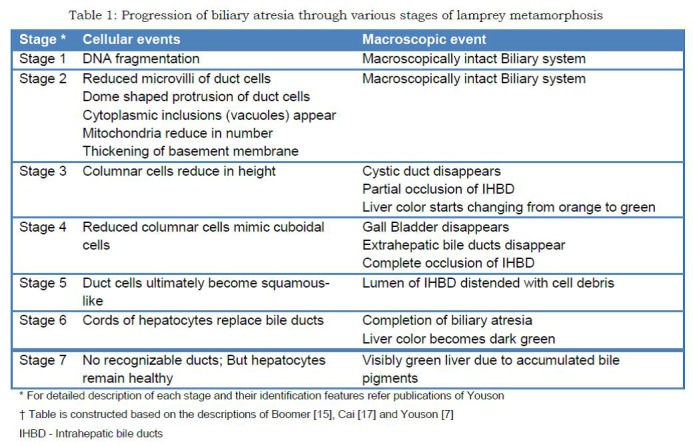
Table 1: Progression of biliary atresia through various stages of lamprey metamorphosis

**Evidence of Adaptation of Lamprey to BA**

 Cai et al [17] found that serum level of bile salt was 13 + 7 µM in larvae, 12 + 10 µM in adult male and 4 + 3 µM in adult females. All these values are within the normal range of lampreys. Level of bile salts in the liver tissue was 2 to 3 times higher in adults than in larvae. This indicates the existence of cholestasis in adult lamprey liver, which is also corroborated histologically by the presence of stainable debris in ductules. It is interesting that serum levels of bile slats are within normal range despite cholestasis in liver. This is possible only if the lampreys have adaptive mechanism and alternate ways of eliminating the bile salts. Extensive research has identified four potential adaptive mechanisms:

1. Reduction and relocation of bile salt synthesis
2. Alteration of bile salt composition
3. Protection of hepatocytes
4. Effective elimination of bile salts by alterative routes

**Adaptive mechanism 1: Reduction or relocation of bile salt synthesis**

 Adult lampreys appear to avoid accumulation of bile salts in plasma by reducing their synthesis. Cyp7A1 is a rate-limiting enzyme in the biosynthesis of bile salts. Recent studies [10, 17] found that mRNA expression of gene encoding this enzyme is 5 to 100 times down regulated in the liver of adult lamprey. On the other hand, expression of this mRNA is increased 100 fold in the gut of adults. These findings indicate that metamorphic lampreys effectively relocate the site of bile salt synthesis from liver to intestine. Greenish discoloration of adult intestine may add support to this assumption. [10] 

**Adaptive mechanism 2: Change in bile salt composition**

 Lampreys have unique bile acid (allocholic acid) and bile alcohol (petromyzonol) in addition to well-known bile acids such as taurocholic acid. The 4 common types of bile salts in lampreys are Petromyzonol sulfate (PZS), Petromyzonamine sulfate (PZN), Petromyzosterol disulfate (PZStD) and 3-keto-petromyzonol sulfate (3kPZS). PZS is the principal bile salt, which has two forms namely C24-PZS and C27-PZS. When human red blood cells are exposed to these bile salts C24-PZS caused more cell lysis than the others [17] Thus C24-PZS is more cytotoxic than C27-PZS and 3kPPZS. Cai et al [17] estimated the relative serum levels of these bile salts in larvae and adults. C24-PZS was 96% in larvae, 72% in adult males and 0% in adult females. C27-PZS was 4% in larvae, 28% in males and 100% in females. This indicates that adult females could effectively alter the bile salt composition by reducing the noxious C24-PZS and increasing the less-toxic C27-PZS. Interestingly, adult males do not follow the same strategy, as the level of C24-C27 ratio is high in them. Instead, adult males convert toxic PZS into non-toxic 3kPZS. The 3kPZS is excreted through the gills of males and they act as sex pheromones to attract ovulating females. [18] Further, bile acids such as allocholic acid, taurocholic acid (TCA), taurochenodeoxycholic acid (TCDCA), and ursodeoxycholic acid (UDCA) were detected in larval liver; but not in adults. [17] Thus adult males and females, by different mechanism, convert toxic bile acids into less toxic bile alcohols. This adaptive change in the composition of bile salts may explain as to why the lamprey livers do not develop cirrhosis despite BA. This chemical change may also facilitate elimination of bile salts through alternative routes. 

**Adaptive mechanism 3: Hepato-protection**

 In addition to reversal of C24-C27 ratio, there appears to be other mechanisms of hepato-protection during metamorphosis. Biliverdin, the typical bile pigment, has antioxidant properties. Increased levels of this pigment can effectively offset the toxic effects of bile salts. [19, 20] Biliverdin is reduced to bilirubin by the enzyme biliverdin reductase (BlvR). mRNA expression of BlvR-A is down-regulated in adult lamprey. Consequently, biliverdin levels are increased in the liver of adult lampreys thereby conferring the hepato-protective effect. This is evident in adult lampreys even macroscopically by the dark green color of the liver. 

**Adaptive mechanism 4: Alternative route of excretion**

 In the absence of biliary ducts, adult lampreys appear to excrete bile salts by 3 alternative routes - namely kidneys, intestine and gills. Excretory pattern is studied by experimentally injecting bromosulfophthalein (BSP), or radioactive taurocholate and tracing their clearance. There is considerable disagreement among the workers as to the major alternative route of bile salt excretion in adult lamprey. 

 Yeh et al [10] showed that the concentration of intravenously injected radioactive taurocholate is 10 times higher in adult gut than in plasma. mRNA expression of HMG-CoA reductase - a rate limiting enzyme in cholesterol synthesis - was also found to be up-regulated in adult gut. But they could not find any trace of radioactive taurocholate in urine thereby indicating that intestine could be the major eliminator of bile salts in the absence of bile ducts. Cai et al [21] originally subscribed this view by noting high levels of H-taurocholic acid in the intestine of adult lampreys. However, they subsequently changed their standpoint when they used BSP clearance studies. [17] They found that 35% of BSP injected is cleared within 72 hours but only 1% of it is eliminated through intestines. Serendipitously one of the lampreys they studied had ureteric obstruction. In this animal the obstructed ureter contained high levels of bile salts and bile pigments. Further when urine was collected by direct cannulation of ureters, urinary BSP clearance was 11% in 24 hours - which approximately matches the 72-hour clearance rate. Further, kidneys undergo extensive structural remodeling during metamorphosis as if they are preparing for a new physiological role. [17] From these observations they concluded that kidneys could be the major route of bile salt and bile pigment elimination in adult lampreys. They also noted urinary excretion of biliverdin was more in males than in females; while urinary bile salt excretion was equal in both sexes. 

As described earlier male lampreys convert PZS into 3kPZS and excrete it through their gills. As 3kPZS is a sex pheromone, the excreted amount of 3kPZS is probably very small and hence it unlikely to be a major mode of bile salt elimination. 

 Conjugated bile salts and bile pigments cannot passively cross cell membrane. Therefore, intestinal or renal excretion of these conjugated salts must be a energy-dependant phenomenon. [21] Indeed, Cai et al [17] studied mRNA expression of various export pumps and cell transport mediators. (Table 2) Although the findings are far from conclusiveness, there is some evidence to believe that bile salts are actively excreted through intestinal and renal epithelium. Additionally, Youson et al [22] have shown that gap junctions and zonulae occludens of hepatocytes disappear during metamorphosis thereby facilitating back diffusion of bile salts and bile pigments from hepatocytes through basolateral membrane. Thus exocrine liver is converted into endocrine liver during metamorphosis. 

**Figure:**
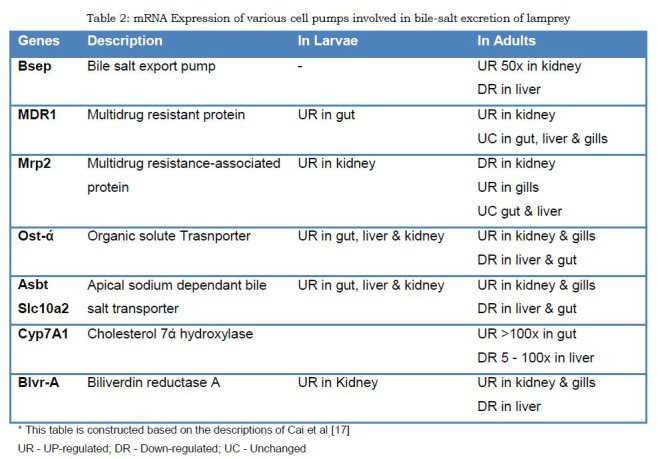
Table 2: mRNA Expression of various cell pumps involved in bile-salt excretion of lamprey

**Level of adaptation in human BA**

 In the background of lessons learnt from lamprey, a closer look at the clinical BA will quickly reveal that these adaptive mechanisms are also present in human beings. Is it not true that phylogeny is repeated in every ontogeny? Like lampreys, hepatic expression of Cyp7A is down regulated in human BA in order to reduce the biosynthesis of bile salts. [23] It is yet to be studied if this enzyme is up-regulated in human gut analogous to lamprey. Athena has frequently witnessed ‘emerald green’ liver in BA. This due to accumulation of biliverdin, which is an obvious attempt of the nature to limit hepato-toxiciy of bile salts. Alternative path of excretion is evident by transplacental excretion of fetal bile products. Effective placental transfer protects the fetal liver in congenital BA, which is why the liver is healthy at birth but subsequently become cirrhotic. In all obstructive jaundice clinicians are familiar with dark yellow urine, which is indicative of compensatory elimination of bile products through kidney. Even active elimination of bile pigments through intestinal epithelium appears to exist in man. In late stages of BA when bilirubin levels exceed 20 mg/dl Athena has seen pale yellow streaky discoloration of stools. However, unlike lampreys, the adaptive mechanisms are incomplete, imperfect and inadequate in human infants, which is why they develop liver damage. Athena calls for focused research to perfect or assist these adaptive mechanisms in human beings so that a radical cure of BA will become feasible. 

**Epilogue**

Athena wonders as to the nature’s purpose of obliterating the well-developed bile ducts in lampreys. Interestingly, in lampreys BA smaller ducts degenerate faster than larger ducts [7], while the reverse of it is true of human BA. Intra-cytoplasmic bile salts accumulates, which are frequently seen in human BA, are absent in lampreys. Athena is not sure of the practical implications of these differences. 

Athena is amused to know that scientists induce metamorphosis of larval lamprey in laboratory by adding anti-thyroid chemicals to the water. [15] Lamprey metamorphosis is thyroxin dependent and hence this technique yields consistently high success to the tune of 99%. If so, does thyroxin has anything to do with the atresia of biliary system? Athena is reminded of a case report wherein Seoud et al [24] described neonatal BA caused by maternal exposure to methimazole. Relation between hypothyroidism and BA is worthy of research.

## Footnotes

**Source of Support:** Nil

**Conflict of Interest:** The author is Editor of the journal. But he did not take part in the evaluation or decision making of this manuscript. The manuscript has been independently handled by two other editors.

